# Involvement of SULF2 in γ-irradiation-induced invasion and resistance of cancer cells by inducing IL-6 expression

**DOI:** 10.18632/oncotarget.7449

**Published:** 2016-02-17

**Authors:** Chan-Hun Jung, Jin-Nyoung Ho, Jong Kuk Park, Eun Mi Kim, Sang-Gu Hwang, Hong-Duck Um

**Affiliations:** ^1^ Division of Radiation Cancer Biology, Korea Institute of Radiological & Medical Sciences, Seoul 01812, Korea; ^2^ Present address: Biomedical Research Institute, Department of Urology, Seoul National University Bundang Hospital, Seongnam 463-707, Korea

**Keywords:** SULF2, IL-6, radiotherapy, cancer invasion, resistance

## Abstract

Cancer cells that survive radiotherapy often display enhanced invasiveness and resistance to death stimuli. Previous findings have suggested that ionizing radiation (IR) induces such undesirable effects by stimulating the STAT3/Bcl-X_L_ pathway. To identify novel cellular components that mediate these actions of IR, we irradiated lung cancer cells with sublethal doses of γ-rays and screened for the induction of IR-responsive genes by microarray analysis. The genes encoding 2 extracellular proteins, SULF2 and IL-6, were found to be upregulated, and these results were confirmed by polymerase chain reactions and western blot analyses. Because the IR-mediated induction of SULF2 was a novel finding, we also confirmed the phenomenon in vivo using xenograft tumors in mice. Analyses of signaling processes revealed that IR induced SULF2 expression via p53, which then promoted IL-6 expression by stabilizing β-catenin, followed by stimulation of the STAT3/Bcl-X_L_ pathway. Consistently, both SULF2 and IL-6 mediated IR-induced invasion and resistance to death stimuli. To investigate whether SULF2 contributes to IR-induced tumor metastasis, we irradiated tumors in mice with sublethal doses of IR. This treatment promoted the entry of tumor cells into the blood stream (intravasation), which was abolished by downregulating SULF2 expression in tumor cells. These results demonstrated that SULF2 can mediate the detrimental effects of IR in vivo. Therefore, SULF2 may be potentially used as a therapeutic and diagnostic target to predict and overcome the malignant effects of IR, particularly in tumors expressing p53 wild-type.

## INTRODUCTION

Although ionizing radiation (IR) is a major therapeutic modality for treating cancer, it can also promote malignant behavior in surviving cancer cells. For example, sublethal doses of IR increase the migratory and invasive potentials of cultured cancer cells [1]. Local irradiation of primary tumors consistently facilitates their distal metastasis in animal models [2-4], suggesting that the failure of local radiotherapy may actually increase morbidity in some instances. Moreover, the cells that survive radiotherapy can also develop secondary resistance to anti-cancer or lethal treatments, a phenomenon termed acquired or adaptive resistance [5, 6]. Therefore, identifying the cellular components involved in such undesirable consequneces of IR is essential for developing new strategies to enhance the efficacy of radiotherapy.

Sublethal doses of IR can activate the transcription factor STAT3, which in turn promotes Bcl-X_L_ expression [7]. Bcl-X_L_ is a pro-survival member of the Bcl-2 protein family [8], and its upregulation by IR was proposed to mediate acquired radioresistance [9]. Although originally identified as key regulators of cell death, certain members of the Bcl-2 family have been recently shown to serve additional roles in regulating cell migration and invasion [10–13]. For example, Bcl-X_L_ has been shown to promote cell invasion and cancer metastasis by stimulating a cellular pathway involving p38 kinase, phosphoinositide 3-kinase, Akt, and matrix metalloproteinase-2 [7, 12]. Therefore, Bcl-X_L_ has also been suggested to mediate IR-induced cell invasion [7]. Given the dual functions of Bcl-X_L_ in mediating the detrimental effects of IR, it is important to determine how IR stimulates the STAT3/Bcl-X_L_ pathway.

Heparin-degrading endosulfatases (SULFs) are extracellular enzymes that remove sulfate groups from heparin sulfate proteoglycans (HSPGs) [14–16]. HSPGs can bind to heparin-binding protein ligands such as cytokines, growth factors, chemokines, and morphogens, and this binding modulates the interactions between ligands and their respective cell-surface receptors. As ligand binding occurs via the sulfate groups of HSPGs, removal of these groups by SULFs also influences receptor-ligand interactions and downstream signaling processes. A relatively well-studied example is the stimulation of the Wnt/β-catenin pathway by SULFs. In this case, sulfated HSPG binds to Wnt, thereby sequestering it from its receptor Frizzled. However, desulfation of HSPG by SULFs results in the release of Wnt from HSPG, subsequent binding of Wnt to Frizzled, and stabilization of the downstream signaling component, β-catenin [14].

Two isoforms of SULF are known, namely SULF1 and SULF2. Although the function of SULF1 remains largely unknown, SULF2 was identified as a potential cancer-driving gene in unbiased screening studies of glioma [17] and breast cancer [18]. SULF2 up-regulation has been consistently observed in various types of cancer, including lung, breast, liver, brain, pancreatic, and gastric cancer [19–24] and was associated with an aggressive tumor phenotype and poor prognosis in patients with hepatocellular carcinoma [21], multiple myeloma [25], and esophageal cancer [26]. Moreover, studies using cancer cells and animal models have shown that SULF2 can facilitate tumor growth and migration [21, 22, 24], supporting its role in tumor formation and progression. However, no information is currently available regarding the possible involvement of SULF2 in radioresponses.

In this study, we performed a microarray analysis to identify novel cellular components that are induced by sublethal doses of IR. We were particularly interested in extracellular, rather than intracellular, components. This preference was based on the view that extracellular components are more attractive therapeutic and diagnostic targets than intracellular components, because certain therapeutic and diagnostic strategies, including the use of neutralizing antibodies and the diagnostic use of blood samples, are more readily applicable to extracellular targets [27]. We found that IR induced SULF2 expression via the p53 transcription factor, which then mediated the ability of IR to stimulate the STAT3/Bcl-X_L_ pathway and increase the cellular invasiveness and survivability against death stimuli. SULF2 exerted these functions by inducing the expression of the pro-inflammatory cytokine IL-6 via β-catenin. We further confirmed the ability of SULF2 to support the detrimental effects of IR in vivo using an animal model. Therefore, SULF2 may be used as an extracellular target to predict and overcome the malignant actions of IR.

## RESULTS

### Microarray analysis of IR-responsive genes in A549 lung cancer cells

To identify novel functional genes that respond to IR, human A549 lung cancer cells were irradiated with γ-rays (10 Gy). These cells were highly resistant to IR and survived the treatment. After 24 h, the irradiated and untreated control cells were analyzed using an Affymetrix HuGeneChip with Robust Multi-chip Analysis (RMA) normalization to select differentially expressed genes. We found 11 and 114 genes that were upregulated (Table 1) and downregulated (Supplementary Table S1), respectively, by >2-fold in the irradiated cells. Among the upregulated genes, 2 encoded the secretory proteins SULF2 and IL-6. IL-6 induction by IR has been previously reported [28, 29], whereas no information is currently available regarding the relationship between IR and SULF2. Therefore, we first sought to investigate the possible role of SULF2 in radioresponses.

**Table 1:** Genes upregulated in A549 lung cancer cells by sublethal doses of γ-irradiation

Gene symbol	Gene ID	Fold change*	Localization	Description
TP53INP1	94241	3.595574	Intracellular	Tumor protein p53 inducible nuclear protein 1
ACTA2	59	2.920948	Intracellular	Homo sapiens actin, alpha 2
MDM2	4193	2.755156	Intracellular	Transformed 3T3 cell double minute 2, p53 binding protein
BTG2	7832	2.723974	Intracellular	Homo sapiens BTG family, member 2
FDXR	2232	2.621785	Intracellular	Ferredoxin reductase
TP53I3	9540	2.468641	Intracellular	Tumor protein p53 inducible protein 3
DGKA	1606	2.34046	Intracellular	Diacylglycerol kinase, alpha 80kDa
CYFIP2	26999	2.24827	Intracellular	Cytoplasmic FMR1 interacting protein
IL6	3569	2.084982	Extracellular	Interleukin 6
SULF2	55959	2.078101	Extracellular	Sulfatase 2
SESN1	27244	2.033297	Intracellular	Sestrin 1

A549 cells were irradiated with 10 Gy of γ-rays. After a 24-h incubation, microarray analysis was performed to compare gene-expression levels in the irradiated and untreated control cells. The table lists genes that showed >2-fold increased expression following irradiation.

* fold-change of gene-expression levels in irradiated cells compared to those observed in untreated cells.

### IR induces SULF2 expression via p53

**Figure 1:**
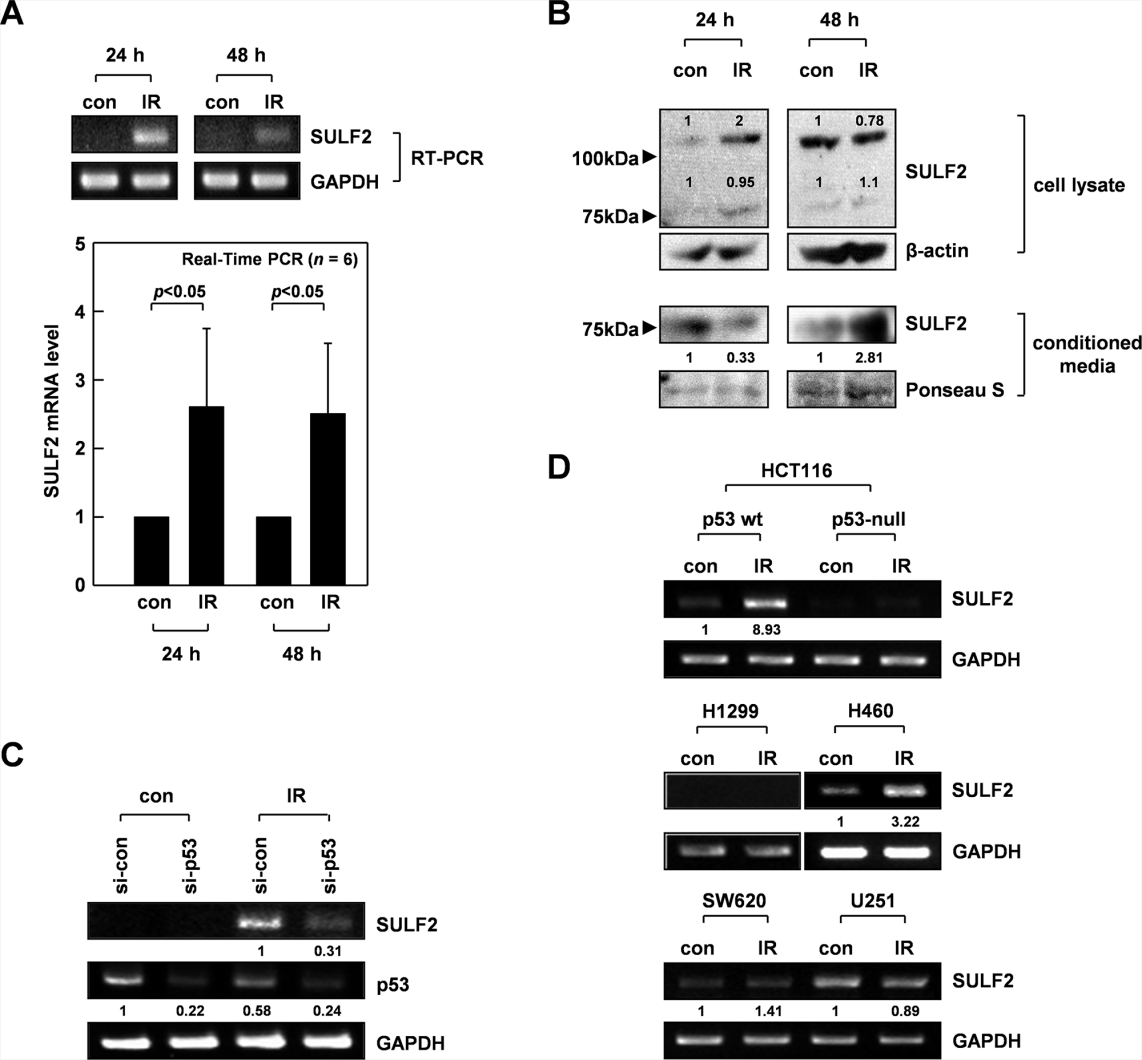
IR induces SULF2 expression via p53 **A.** A549 cells were irradiated with γ-rays (10 Gy). After the indicated times, SULF2 mRNA levels in irradiated and untreated control cells were compared by RT-PCR (top) or real-time PCR (bottom). The real-time PCR data are presented as relative values normalized to those of the internal control (GAPDH). The graphs show the means and standard deviations from 6 independent experiments. **B.** Cell lysates and conditioned media were prepared at the indicated times after irradiation. SULF2 protein levels were analyzed by western blotting. β-actin was used as a loading control for cell lysates, and protein loading with the conditioned media was verified by Ponceau S staining. **C.** A549 cells were treated with control or p53-targeting siRNA. After a 24-h recovery, the cells were irradiated with 10 Gy of γ-rays and incubated for an additional 24 h. The mRNA levels of SULF2 and p53 were compared by RT-PCR. **D.** The indicated cells were irradiated with 10 Gy of γ-rays. After a 24-h incubation, RT-PCR was performed to compare SULF2 mRNA levels in irradiated and untreated control cells.

Reverse transcription-polymerase chain reaction (RT-PCR) and real-time PCR analyses further confirmed that 10 Gy of γ-rays increased SULF2 mRNA levels in A549 cells by >2-fold, which was evident at 24-48 h after irradiation (Figure 1A). Western blot analysis of SULF2 protein expression revealed 2 bands of ∼125 kDa and ∼75 kDa, corresponding to a pro-form and processed form, respectively, of SULF2 (Figure 1B) [30]. Only the 75-kDa band was detected in the conditioned media of cells, as reported previously [30]. IR elevated the levels of both the 125- and 75-kDa forms in cell lysates, which was evident at 24 h, but not at 48 h, post-irradiation. The loss of the IR effect at 48 h might reflect the secretion of cellular SULF2 protein. Indeed, IR increased the levels of the 75-kDa form in conditioned media at 48 h, but not at 24 h. Collectively, these data suggest that IR treatment increased SULF2 mRNA and protein levels, which was followed by an extracellular accumulation of the processed forms of the SULF2 protein.

SULF2 is a transcriptional target of p53 [31]. Considering that A549 cells express wild-type p53 and that p53 is a major transcription factor stimulated by IR [32], we hypothesized that IR might induce SULF2 expression via p53. Indeed, the ability of IR to increase SULF2 mRNA levels was markedly attenuated by small interfering RNA (siRNA)-mediated knockdown of p53 expression in A549 cells (Figure 1C). To further confirm the role of p53, we utilized HCT116 colon cancer cells expressing wild-type p53 or having a p53-null mutation [33]. IR increased SULF2 mRNA levels only in the p53-expressing, but not in the p53-null, HCT116 cells (Figure 1D). IR consistently increased SULF2 expression in H460 (wild-type p53), but not H1299 (p53-null) lung cancer cells. Moreover, IR failed to increase SULF2 expression in SW620 colon cancer cells and U251 glioma cells expressing a mutant p53 lacking DNA-binding activity [34, 35]. These results demonstrated that IR can induce SULF2 expression in multiple types of cancer cells and that p53 is a major transcription factor mediating this effect. However, it should be noted that SW620 and U251 cells expressed SULF2 mRNA constitutively, although the expression level was not further increased in response to IR. This suggests that p53 is not solely responsible for the expression of SULF2.

### SULF2 mediates IR-induced invasion

**Figure 2:**
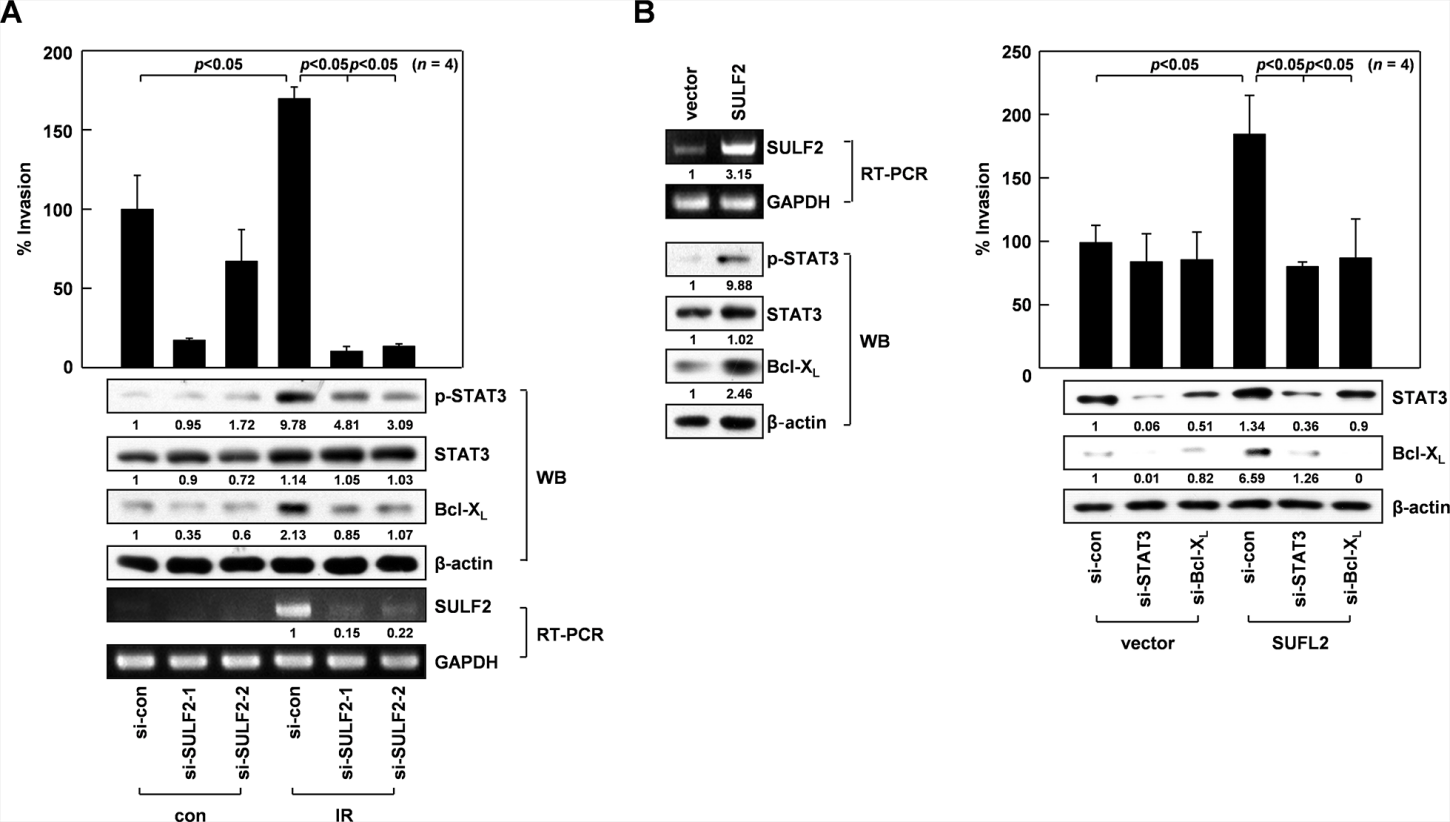
SULF2 mediates IR-induced cell invasion **A.** A549 cells treated with control RNA or 2 sets of SULF2 siRNA were irradiated with 10 Gy of γ-rays. After a 24-h incubation, cell invasiveness was compared on Matrigel-coated polycarbonate filters (top). The levels of STAT3, phosphorylated STAT3, Bcl-X_L_, and SULF2 were compared by western blotting (WB) or RT-PCR (bottom). **B.** *Left,* A549 cells were transfected with the empty pcDNA3 vector or the vector encoding SULF2. After a 48-h incubation, the levels of SULF2, STAT3, phosphorylated STAT3, and Bcl-X_L_ were compared by RT-PCR or western blotting. *Right,* Cells were transfected with expression vectors and siRNAs in the indicated combinations, and cellular invasiveness and protein levels of STAT3 and Bcl-X_L_ were compared.

We previously showed that sublethal doses (10 Gy) of IR promote A549 cell invasion by stimulating the STAT3/Bcl-X_L_ pathway [7]. The abilities of IR to increase STAT3 phosphorylation, Bcl-X_L_ levels, and cellular invasiveness were all abolished or attenuated by silencing SULF2 mRNA expression using 2 different siRNAs (Figure 2A), suggesting that IR promotes STAT3/Bcl-X_L_ pathway activation and cell invasion in a SULF2-dependent manner. SULF2 overexpression in A549 cells consistently increased STAT3 phosphorylation, Bcl-X_L_ levels, and cellular invasiveness (Figure 2B). Moreover, SULF2-induced invasion was prevented by knocking down STAT3 or Bcl-X_L_, confirming that SULF2 promotes cell invasion by stimulating the STAT3-Bcl-X_L_ pathway. Overall, these results demonstrated that SULF2 mediates IR-induced invasion by activating the STAT3/Bcl-X_L_ pathway.

### SULF2 mediates IR-induced IL-6 expression via β-catenin

**Figure 3:**
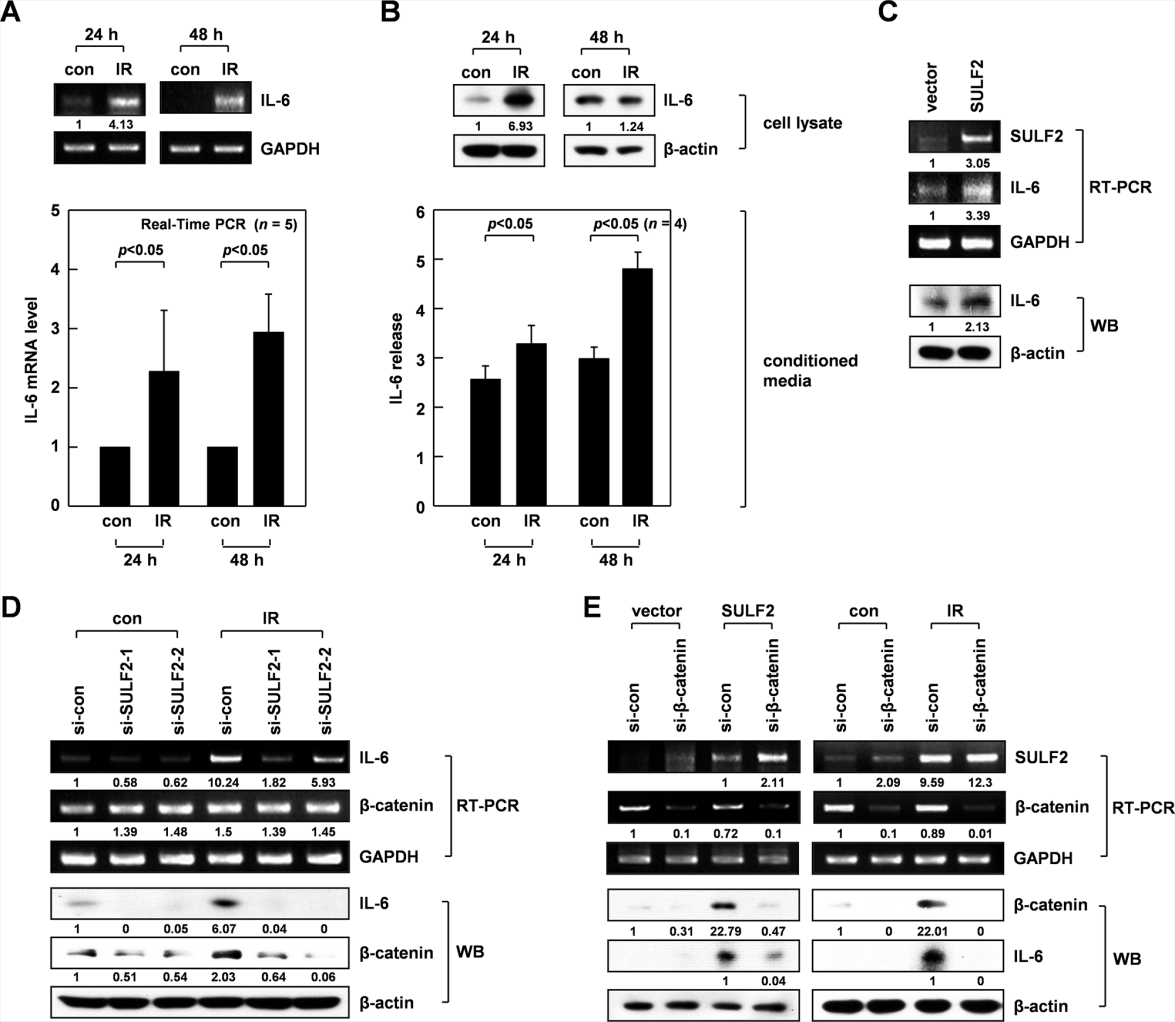
SULF2 mediates IR-induced IL-6 expression via β-catenin **A.** The irradiated and untreated control cells were analyzed for IL-6 mRNA expression by RT-PCR (top) or real-time PCR (bottom). **B.** Cell lysates and conditioned media from the irradiated and untreated control cells were prepared. IL-6 protein levels were analyzed by western blotting (cell lysates) and ELISA (conditioned media). **C.** Control and SULF2 A549 transfectants were analyzed for their levels of SULF2 and IL-6 expression by RT-PCR and western blotting. **D.** Cells treated with control or SULF2 siRNAs were irradiated and analyzed for IL-6 and β-catenin expression by RT-PCR and western blotting. **E.** Cells were transfected with SULF2 expression vectors and β-catenin siRNAs using the indicated combinations (left panels). Alternatively, cells were treated with control or β-catenin siRNA and irradiated (right panels). In both cases, SULF2, β-catenin, and IL-6 expression levels were analyzed by RT-PCR or western blotting.

The results from RT-PCR and real-time PCR experiments also confirmed the ability of IR to increase IL-6 mRNA levels in A549 cells, as observed 24-48 h after irradiation (Figure 3A). IR consistently elevated the cellular levels of IL-6 protein at 24 h, and its accumulation in conditioned media was particularly evident at 48 h (Figure 3B). These data suggested that IR increased IL-6 secretion by stimulating its expression. Increases in IL-6 mRNA and protein levels were also observed upon SULF2 overexpression (Figure 3C), indicating the ability of SULF2 to induce IL-6 expression. Moreover, IR-induced increases in IL-6 mRNA and protein levels were attenuated by SULF2 knockdown (Figure 3D), suggesting that SULF2 mediates IR-induced IL-6 expression.

SULF2 can modulate the signaling processes of various cell-surface receptors, and activation of the Wnt/β-catenin pathway is one such example [14]. Given the ability of β-catenin to positively regulate IL-6 expression under other experimental settings [36], we investigated whether β-catenin was involved in the IL-6 expression induced by IR and SULF2. Indeed, IR (Figure 3D) or SULF2 overexpression (Figure 3E,) left panels) increased β-catenin protein expression in A549 cells, but not that of β-catenin mRNA, suggesting that stabilization of the β-catenin protein occurred. Such effects of IR were abolished by SULF2 downregulation (Figure 3D), suggesting that IR stabilizes β-catenin via SULF2. Moreover, the knockdown of β-catenin attenuated the IL-6 accumulation induced by SULF2 overexpression (Figure 3E, left panels) or IR (Figure 3E, right panels). Collectively, these data indicated that IR-induced SULF2 leads to increased IL-6 expression by stabilizing β-catenin.

### IL-6 mediates SULF2-induced cell invasion

**Figure 4:**
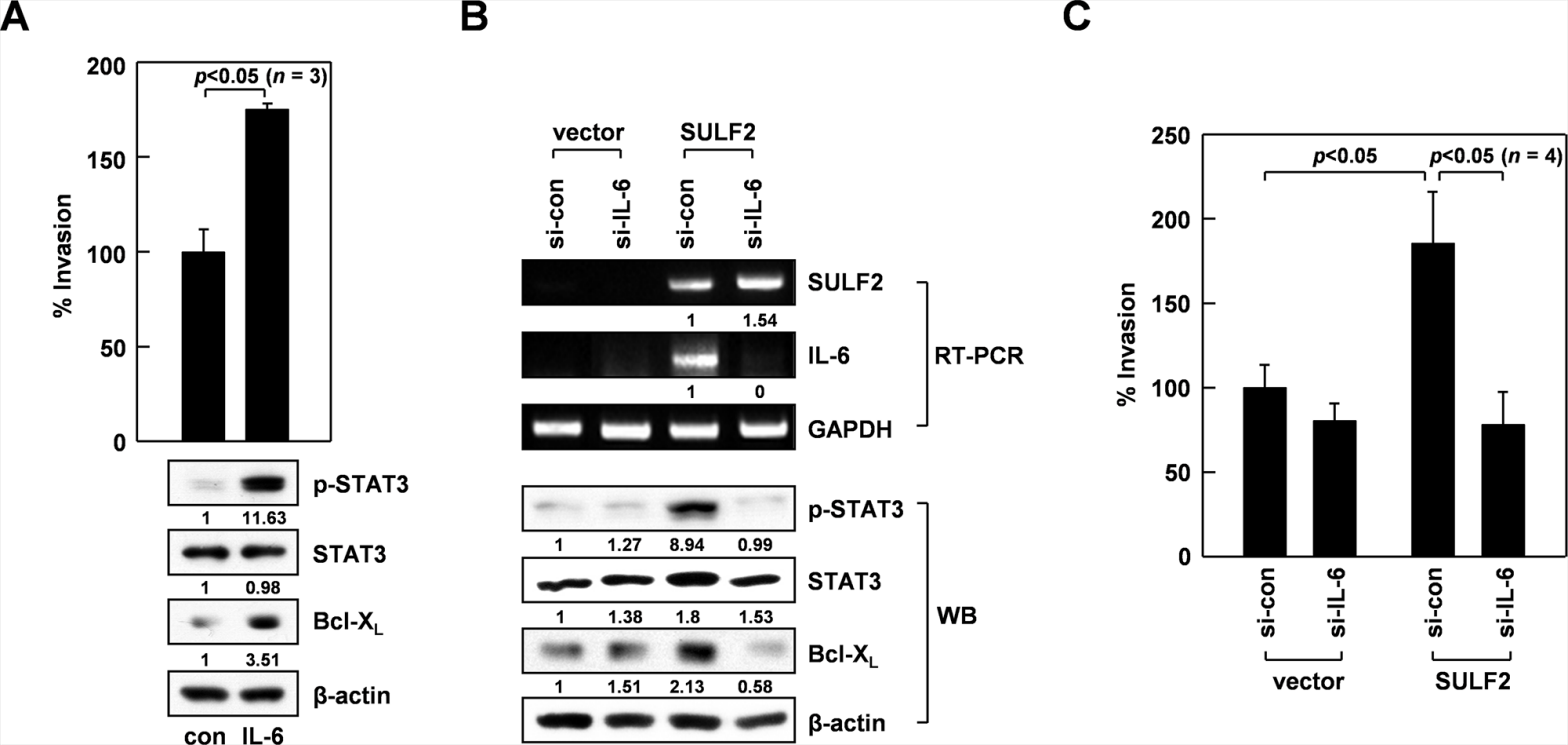
IL-6 mediates the ability of SULF2 to stimulate the STAT3-Bcl-XL pathway **A.** A549 cells were incubated in the presence or absence of IL-6 (20 ng/mL) for 24 h. Cell invasiveness and the levels of indicated proteins were compared. **B.** The cells were transfected with the SULF2 expression vector and IL-6 siRNAs using the indicated combinations. Cellular levels of the indicated components were analyzed by RT-PCR or western blotting. **C.** Invasiveness of the transfectants was compared.

IL-6 can stimulate STAT3 [37]. Thus, we treated A549 cells with IL-6 to confirm this effect under our experimental conditions. As expected, IL-6 exposure resulted in increased STAT3 phosphorylation, Bcl-X_L_ levels, and cellular invasiveness (Figure 4A), demonstrating the ability of IL-6 to stimulate the STAT3/Bcl-X_L_-dependent invasion pathway. Moreover, IL-6 knockdown abolished the ability of SULF2 to promote STAT3 phosphorylation, Bcl-X_L_ accumulation (Figure 4B), and cell invasion (Figure 4C), indicating that SULF2 stimulates the STAT3/Bcl-X_L_-dependent invasion pathway by inducing IL-6.

### SULF2 responds to IR *in vivo*

**Figure 5:**
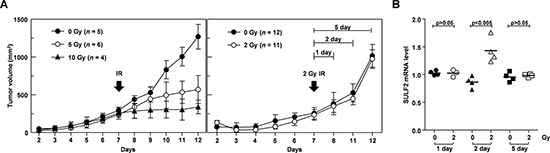
Sublethal doses of IR increase SULF2 mRNA levels *in vivo* **A.** H460 cells were implanted to form xenograft tumors in mice. On the 7th day after implantation, the tumors were locally irradiated with the indicated doses of γ-rays. Tumor diameters were measured, and tumor volumes were calculated on the specified days. **B.** At 1, 2, or 5 days post-irradiation, the mice were sacrificed and the tumors were removed. SULF2 mRNA levels in the tumors were compared by RT-PCR. The values shown represent the relative levels of SULF2 mRNA normalized to those of GAPDH.

To analyze the expression and function of SULF2 *in vivo*, we used H460 lung cancer cells, because these cells efficiently form tumors in mice [12, 38], and we confirmed that SULF2 in H460 cells responds to IR (Figure 1D). The growth of tumors in mice was not significantly influenced by exposure to 2 Gy of IR, but was significantly reduced using IR doses of 5 Gy or higher (Figure 5A). Therefore, a dose of 2 Gy was chosen to further investigate the role of SULF2 in IR-induced cell invasion in mouse models. We first confirmed that an IR dose of 2 Gy increased SULF2 expression in cultured H460 cells (see below). The same dose consistently increased SULF2 mRNA levels in xenograft tumors in mice, as determined at 2 days post-irradiation (Figure 5B). These results demonstrated the ability of IR to induce SULF2 expression *in vivo*. However, the effect of IR disappeared 3 days after irradiation, indicating that SULF2 responds to IR transiently.

### SULF2 mediates IR-induced intravasation of cancer cells

**Figure 6:**
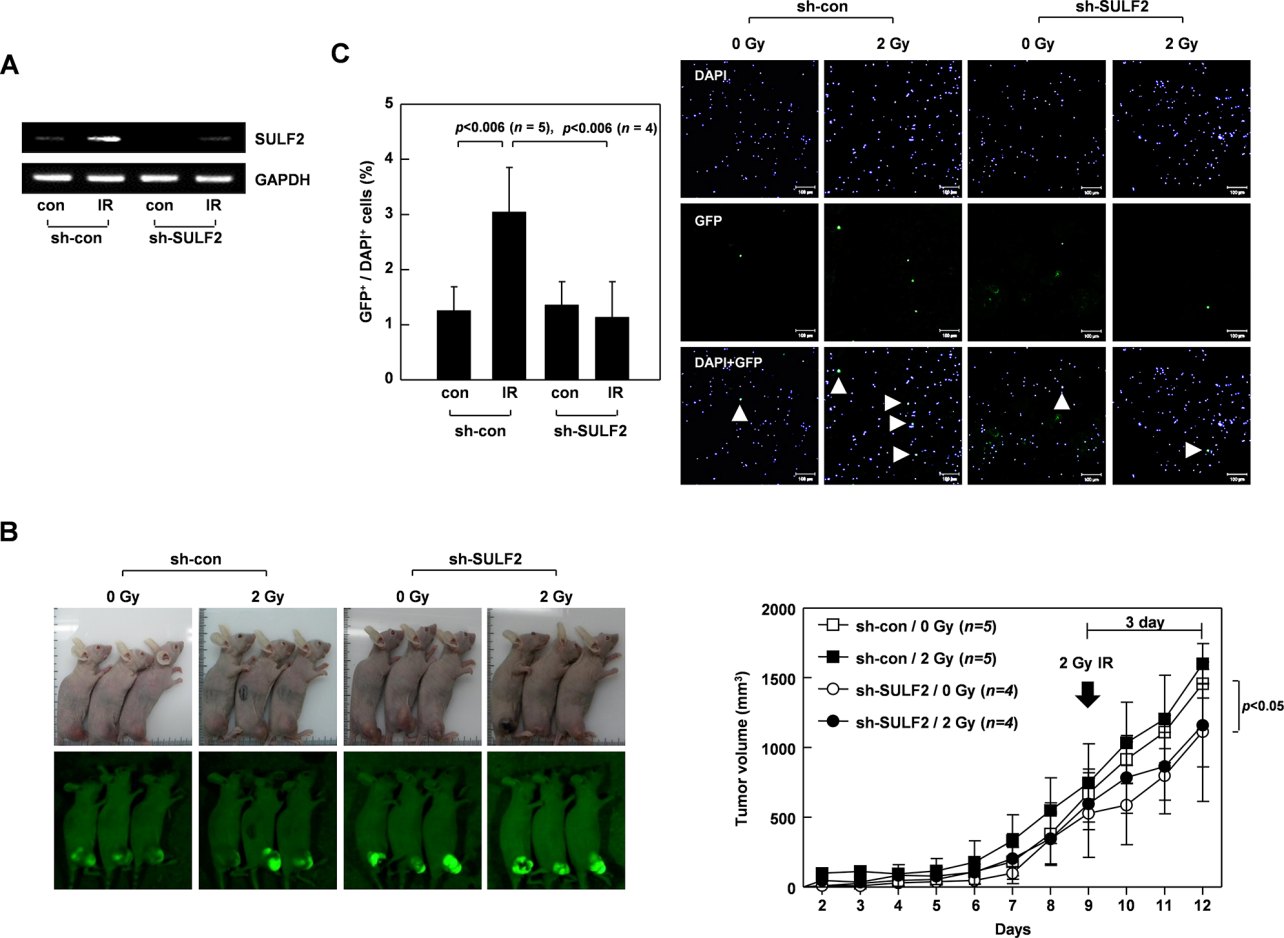
SULF2 mediates the IR-induced intravasation of tumor cells **A.** H460 cells transfected with the pEGFP-C1 vector were infected with a lentivirus expressing control or SULF2-specific shRNA. These double transfectants were irradiated with 2 Gy of γ-rays. After 24 h, SULF2 mRNA levels were compared by RT-PCR. **B.** The double transfectants were injected into the hind legs of mice to form xenograft tumors. Tumors were locally irradiated with 2 Gy of γ-rays at day 9. Tumor volumes were calculated on the indicated days. The photograph shown was taken using a Nikon Coolpix L100 digital camera, and the green (GFP) images were captured under blue light using an IF550 emission filter (Olympus). **C.** At 3 days post-irradiation, blood was obtained from the mice. Blood cells were stained with DAPI and analyzed by confocal microscopy. Circulating tumor cells were identified as GFP- and DAPI-positive cells (arrow). The graphs show the tumor-cell/DAPI-positive-cell ratio for each blood sample.

To investigate whether SULF2 mediates the pro-invasive activity of IR *in vivo*, we utilized our previously established mouse model to compare the intravasation potential of cancer cells, a property that relies on their invasive activity [12, 38]. For this assay, H460 cells transfected with a green-fluorescence protein (GFP)-expression vector were infected with lentiviral particles expressing control or SULF2-specific short hairpin RNA (shRNA). We confirmed that the introduction of SULF2 shRNA abolished the IR-induced increase in SULF2 expression (Figure 6A). The transfectants were injected into the hind legs of mice to form tumors (Figure 6B, left). The growth of these tumors was slightly retarded by the expression of SULF2 shRNA (Figure 6B, right), consistent with a previous report that SULF2 facilitates cell growth [21]. However, as confirmed in the preliminary experiments (Figure 5A), irradiation of the tumors with 2 Gy of IR did not significantly influence their growth. This was true regardless of the expression of SULF2 shRNA. The intravasation potential of the tumors was compared by analyzing the appearance of GFP-expressing tumor cells in mouse blood by confocal microscopy 3 days after irradiation (Figure 6C). The number of circulating tumor cells was not significantly altered by the expression of SULF2 shRNA. Therefore, the slight decrease in tumor size caused by SULF2 shRNA did not appear to significantly influence the number of circulating tumor cells. However, the number of circulating tumor cells was markedly increased when the tumors were irradiated with 2 Gy of IR, suggesting that sublethal doses of IR promote the intravasation of tumor cells. Importantly, this effect of IR was prevented by expressing SULF2 shRNA in tumor cells, supporting the possibility that SULF2 mediates the ability of IR to promote cancer cell invasion *in vivo*.

### SULF2 and IL-6 mediate IR-induced resistance to death stimuli

**Figure 7:**
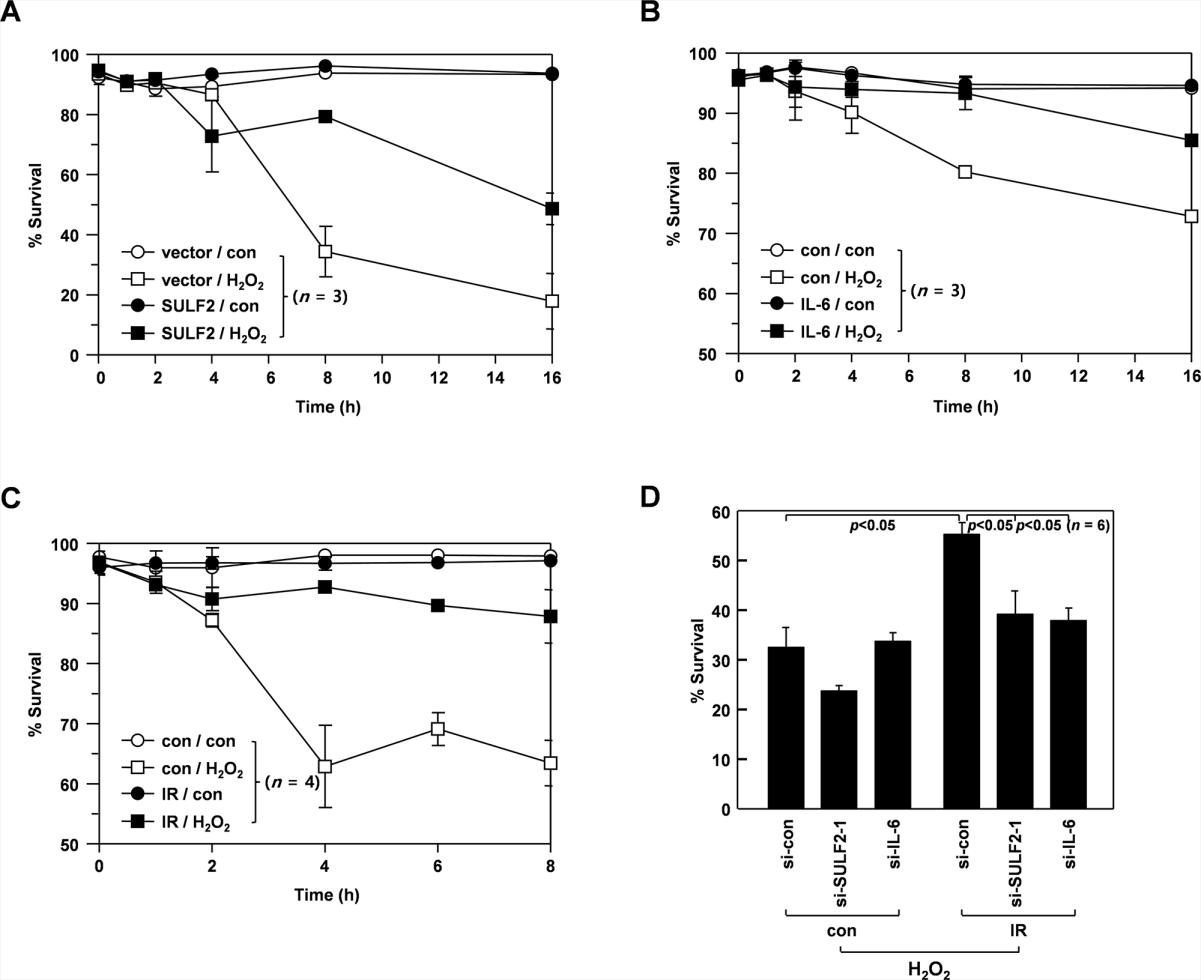
SULF2 and IL-6 mediate IR-induced resistance to death stimuli **A.** H460 cells were transfected with pcDNA3 or pcDNA3/SULF2. After a 48-h recovery, the transfectants were treated with or without 320 μM H_2_O_2_ for the indicated periods. Cellular viability was analyzed by flow cytometry. **B.** H460 cells incubated in the presence or absence of IL-6 (20 ng/mL) for 24 h were treated with or without 320 mM H_2_O_2_ for the indicated periods, and cellular viability was analyzed. **C.** H460 cells were irradiated with γ-rays (2 Gy). After a 48-h incubation, irradiated and untreated control cells were challenged with or without 320 μM H_2_O_2_ for the indicated periods, and analyzed for viability. **D.** H460 cells were treated with the specified siRNAs for 24 h, after which they were irradiated with 2 Gy of γ-rays. After a 48-h recovery, the irradiated and untreated control cells were challenged with 320 μM H_2_O_2_ for 8 h, and analyzed for viability.

Considering the ability of SULF2 and IL-6 to increase the cellular levels of Bcl-X_L_, it is likely that SULF2 and IL-6 might contribute to the IR-induced resistance to death stimuli. To investigate this possibility, we induced cell death by treating H460 cells with H_2_O_2_ (320 mM). Cell death was attenuated by SULF2 overexpression (Figure 7A) or IL-6 pretreatment (Figure 7B). Interestingly, H_2_O_2_-induced cell death was also dramatically reduced when the cells were irradiated with sublethal doses (2 Gy) of IR at 24 h prior to H_2_O_2_ treatment (Figure 7C). This finding demonstrated the ability of IR to induce resistance to death stimuli. However, the protective effect of IR was abolished when SULF2 or IL-6 expression was knocked down (Figure 7D), supporting their involvement in IR-induced resistance.

## DISCUSSION

In this study, we showed that IR promotes the expression and extracellular accumulation of SULF2. This was initially suggested by our microarray analysis and was further confirmed by RT-PCR, real-time PCR, and western blot analyses. Using p53-null and p53-silenced cells, we also verified that p53 mediates IR-induced SULF2 expression. As SULF2 induction was observed using a sublethal dose of IR, which promotes cell invasion, we subsequently investigated the possible involvement of SULF2 in IR-induced cell invasion. Indeed, we found that SULF-2 knockdown attenuates the ability of IR to stimulate the STAT3/Bcl-X_L_ pathway and cell invasion, and that SULF2 overexpression mimics the pro-invasive effects of IR. Therefore, it is clear that SULF2 mediates IR-induced cell invasion by stimulating the STAT3/Bcl-X_L_ pathway. Importantly, this function of SULF2 appears to also operate *in vivo*, as we further demonstrated that sublethal doses of IR increased SULF2 expression in tumor cells in mice and also promoted their intravasation in a SULF2-dependent manner.

The microarray, PCR, western blot, and ELISA analyses also revealed that IR induced the expression and extracellular accumulation of IL-6 under our experimental conditions. By treating cancer cells with exogenous IL-6, we confirmed that IL-6 promotes cell invasion by stimulating the STAT3/Bcl-X_L_ pathway. Given that SULF2 and IL-6 stimulate the same invasion pathway, it was important to determine their hierarchical and functional relationship. Our data indicated that SULF2 acts upstream of IL-6 in IR-induced signaling. First, IR-induced IL-6 expression was prevented by SULF2 knockdown. Second, SULF2 overexpression induced IL-6 expression, highlighting the ability of SULF2 to induce IL-6. Moreover, SULF2 overexpression failed to stimulate the STAT3/Bcl-X_L_-dependent invasion pathway in IL-6-knockdown cells, indicating that IL-6 acts as a downstream mediator of SULF2-induced invasion.

To determine the cellular components involved in SULF2-induced IL-6 expression, we focused on β-catenin. This decision was based on a previous report showing that SULF2 can stimulate the β-catenin pathway [14] and that β-catenin can induce IL-6 expression [36]. We found that IR increased β-catenin protein expression in a SULF2-dependent manner, and that SULF2 overexpression required β-catenin to induce IL-6. These data suggest that IR-induced SULF2 induces IL-6 expression via β-catenin. Taken together, our findings suggest that IR promotes cell invasion by inducing the signaling pathway that sequentially involves p53, SULF2, β-catenin, IL-6, STAT3, and Bcl-X_L_.

We believe that this pathway is also responsible for the IR-induced cellular resistance to death stimuli. The cytoprotective functions of SULF2 and IL-6 were initially suggested by the observation that SULF2 overexpression or IL-6 treatment protected cells from lethal concentrations of H_2_O_2_. To directly confirm their roles in IR-induced resistance, we established an experimental condition in which pre-irradiation with sublethal doses of IR conferred cells with resistance to subsequent challenges with H_2_O_2_. Such a protective effect of IR was abolished by knocking down either SULF2 or IL-6, thereby supporting their roles in IR-induced resistance.

**Figure 8:**
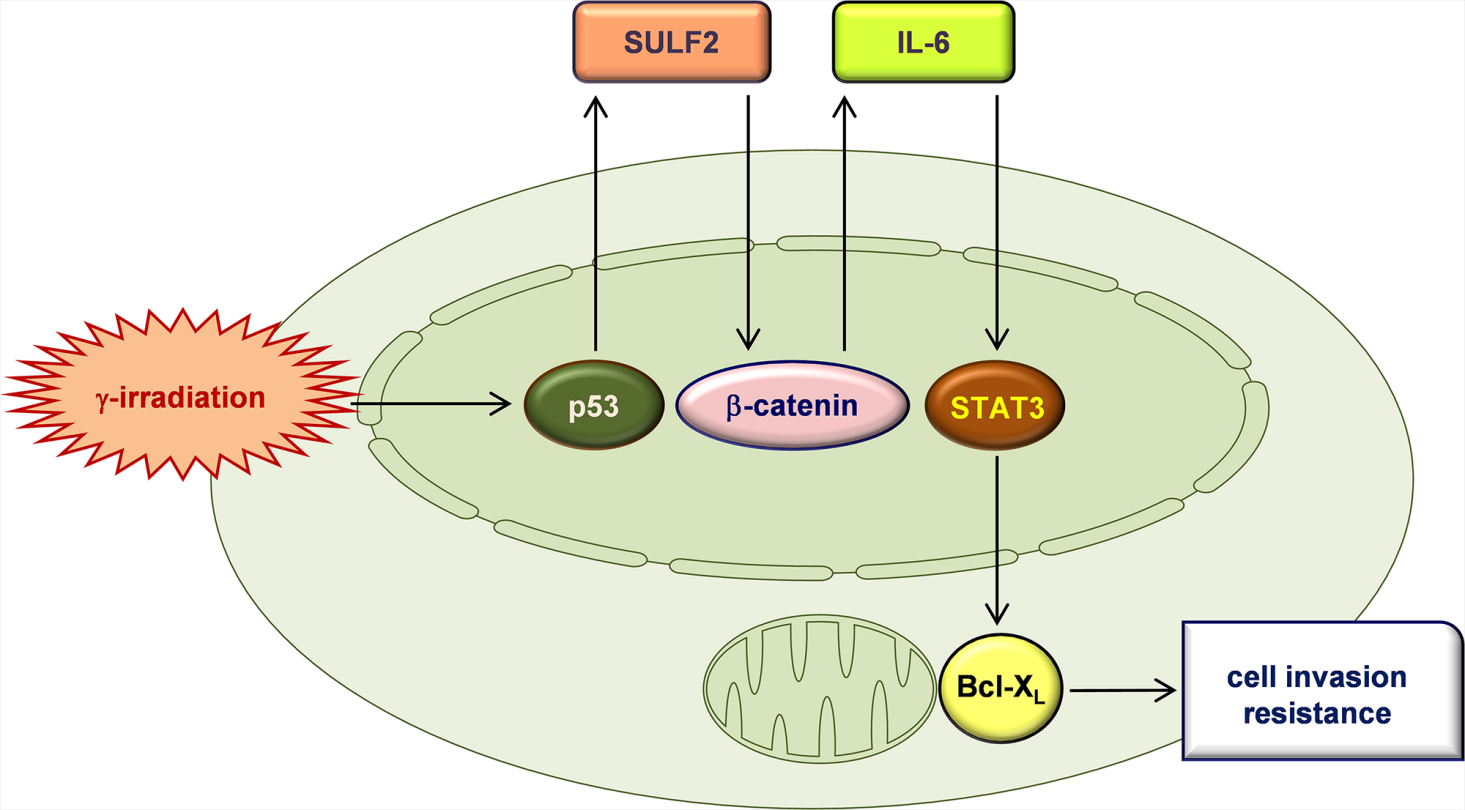
A proposed model for the IR-induced signaling pathway that leads to an increase in cellular invasiveness and resistance IR induces the expression of SULF2 via p53. SULF2 is externally secreted, where it stimulates the β-catenin pathway, promoting the expression and secretion of IL-6. IL-6 then stimulates the STAT3-Bcl-X_L_ pathway, which leads to an increase in cellular invasiveness and resistance.

Figure 8 shows a schematic description of our main findings. To the best of our knowledge, this is the first demonstration of the role of SULF2 in radioresponse: SULF2 mediates the malignant actions of IR to induce cancer cell invasion, intravasation, and resistance. These newly identified functions of SULF2 may contribute to tumor progression following the failure of radiotherapy, particularly in the case of tumors expressing p53 wild-type. This latter view is based on the role of p53 in IR-induced SULF2 expression. This role for p53 contrasts with its canonical tumor-suppressing functions such as inhibiting cell invasion and inducing cell death [39]. However, consistent with our findings, p53 mediated the IR-induced expression of matrix metalloproteinase-2, a key enzyme involved in cell invasion, in osteosarcoma and colon cancer cells [40]. Moreover, like other cellular signaling molecules [41], p53 can induce both cell death and cell survival pathways in response to lethal stimuli, including IR [42]. Our findings suggest that the survival pathway is predominantly induced when cells are irradiated with sublethal doses of IR.

In conclusion, we showed that SULF2 is a novel secretory protein that responds to IR and increases cellular invasiveness and resistance. Therefore, the SULF2 pathway may be used as a diagnostic and therapeutic target for predicting and overcoming the malignant actions of IR.

## MATERIALS AND METHODS

### Antibodies and recombinant proteins

The following antibodies were used in this study: anti-SULF2, anti-IL-6, and anti-β-catenin from Santa Cruz Biotechnology (Santa Cruz, CA, USA); anti-Bcl-X_L_, anti-STAT3, and anti-phospho-STAT3 from Cell signaling Technology (Danvers, MA, USA); anti-β-actin from Sigma-Aldrich (St. Louis, MO, USA). Recombinant human IL-6 was purchased from Millipore (Bedford, MA, USA).

### siRNAs

siRNAs targeting SULF2, p53, IL-6, and Bcl-X_L_ were purchased from Ambion (Austin, TX, USA). For SULF2, two sets of siRNAs were obtained and numbered si-SULF2-1 and -2. Their catalogue numbers were as follows: si-SULF2-1 (121056), si-SULF2-2 (121058), si-p53 (s605), si-IL-6 (s7312), si-Bcl-X_L_ (120717). Santa Cruz Biotechnology provided siRNAs targeting β-catenin (sc-44275) and STAT3 (sc-29209).

### Cell culture, transfection, and treatment

All cells used in this study were obtained from the American Type Culture Collection (Rockville, MD, USA), except for HCT116 colon cancer cells (a generous gift from Dr. Bert Vogelstein) and U251 glioma cells (Cell Lines Service; Eppelheim, Germany). Cells were cultured in RPMI-1640 (A549, H1299, and H460 lung cancer cells) or DMED (HCT116, SW620, and U251 cells) medium supplemented with 10% heat-inactivated FBS. SULF2 expression constructs were prepared using the pcDNA3 vector (Invitrogen, Carlsbad, CA, USA). Expression vectors and siRNAs were introduced into cells using Lipofectamine 2000 (Invitrogen), according to the manufacturer's protocol. The transfectants were used for the indicated experiments after 24-48 h of recovery. For irradiation, cells (3 × 10^5^) were seeded into 60-mm dishes, grown until they reached 70-80% confluence, and exposed to γ-rays from a ^137^Cs γ-ray source (Atomic Energy of Canada, Mississauga, Canada) at a dose rate of 3 Gy/min. Alternatively, the cells were treated with the indicated concentrations of IL-6 or H_2_O_2_.

### DNA microarray analysis

A549 cells were irradiated with 10 Gy of γ-rays and grown for 24 h. Subsequently, total RNA was prepared from irradiated and untreated control cells using the Hybrid-R^TM^ Kit (GeneAll, Seoul, Korea). Complementary DNA was synthesized from 2 μg total RNA using M-MLV Reverse Transcriptase and Oligo(dT)15 primers (Promega, Madison, WI, USA) and used for DNA microarray analysis of gene expression, which was performed by ISTECH (Seoul, Korea) using a GeneChip® HuGene-1_0-st-v1 (Affymetrix, Santa Clara, CA). The data were analyzed with the RMA algorithm and Affymetrix Expression Console (Version 1.1). Genes found differentially expressed by >2-fold (*P* < 0.05) were selected and analyzed via the Gene Ontology database (Version 2007-02).

### RT-PCR and quantitative real-time PCR

RT-PCR was performed by amplifying cDNA in Premix PCR solution (Takara, Shiga, Japan) with the following primers: SULF2, 5′-GAA AAG AGG CAG ATT CAC GTC GTT TCC AG-3′ and 5′-ATC TGG TGC TTC TTT TGG GAT GCG GGA G-3′; IL-6, 5′-TCT GGA TTC AAT GAG GAG AC-3′ and 5′-TGA GAT GAG TTG TCA TGT CC-3′; GAPDH, 5′-CAT CTC TGC CCC CTC TGC TGA-3′ and 5′-GGA TGA CCT TGC CCA CAG CCT-3′. Quantitative real-time PCR was performed using the SYBR Fast Universal qPCR Kit (Kapa Biosystems, Woburn, MA, USA) and the following primers: SULF2, 5′-CCT CTT CCC AAA CGC ATC TC-3′ and 5′-GCG CAT GAT CCA GTG TTT GT-3′; IL-6, 5′-AGT GGC TGC AGG ACA TGA CAA-3′ and 5′-CAA TCT GAG GTG CCC ATG CTA-3′; GAPDH, 5′-CAT CTC TGC CCC CTC TGC TGA-3′ and 5′-GGA TGA CCT TGC CCA CAG CCT-3′. The results of RT-PCR and real-time PCR amplifications were analyzed by agarose gel electrophoresis and an IQ-5 Real-Time System (Bio-Rad, Hercules, CA, USA), respectively. GAPDH was used as the internal control for normalization purposes.

### Western blot analysis

Cell lysates were prepared by incubating cells on ice for 30 min in a buffer containing 20 mM Tris-HCl (pH 7.4), 100 mM NaCl, 0.5% NP-40, 0.1 mM Na_3_VO_4_, 50 mM NaF, 30 mM Na_4_O_7_P_2_·10H_2_O, and a protease inhibitor cocktail (GenDEPOT, Barker, TX, USA). Conditioned media were collected and concentrated using Amicon Ultra 30K MWCO centrifugal filter devices (Millipore, Bedford, MA, USA). Proteins in the cell lysates and conditioned media were separated by SDS-PAGE, electrotransferred to nitrocellulose filters (Millipore), and analyzed using the specified antibodies and an ECL detection system (Thermo Scientific, Rockford, IL, USA). Where indicated, proteins in the filters were stained with Ponceau S to confirm equal sample loading.

### ELISA of IL-6

IL-6 levels in conditioned media were analyzed using the IL-6 High Sensitivity Human ELISA Kit (Abcam, Cambridge, UK), according to the manufacturer's instructions.

### Invasion assay

Invasion assays were performed as described previously [43]. Briefly, cells in serum-free media were seeded onto the upper surfaces of Matrigel-coated Transwell chambers (BD Biosciences, Bedford, MA, USA). The lower compartments of the chambers were filled with medium supplemented with 10% heat-inactivated FBS. After a 16-h incubation, the cells that invaded the lower surface of the filter were stained using a Diff-Quick Kit (Fisher Scientific, Pittsburgh, PA, USA) and counted under a microscope.

### Cellular viability analysis

Cells were stained with propidium iodide (5 μg/mL), and analyzed by flow cytometry to monitor their staining intensities and sizes. Cells displaying high permeability to propidium iodide or a reduced size were considered dead, as previously defined [44].

### Animals

Six-week-old BALB/cAnNCrj-nu/nu mice (Charles River, Wilmington, MA, USA) were employed in this study using protocols approved by our Institutional Animal Care and Use Committee.

### Analysis of tumor growth and SULF2 expression *in vivo*

H460 cells were subcutaneously injected (3 × 10^6^/mouse) into the hind legs of mice to form xenograft tumors. Tumor volumes were calculated as described previously.^45^ When the tumors reached a size of ∼200 mm^3^, they were locally irradiated with the specified doses of γ-rays from a ^60^Co γ-IR source (Theratrom 780; AECL Ltd., Mississauga, Ontario, Canada). At 1, 2, and 5 days post-irradiation, the mice were anesthetized and the tumors were surgically removed. Total RNA was prepared from the tumors using the Hybrid-RTM Kit (GeneAll). The levels of SULF2 mRNA expression in tumors were analyzed by RT-PCR, as described above.

### Intravasation assay

GFP-expressing cells were obtained by transfecting H460 cells with the pEGFP-C1 vector (Clontech, Mountain View, CA, USA) and selecting them in 1 mg/mL of G418 sulfate (Santa Cruz Biotechnology). These transfectants were infected with lentivirus particles expressing control or SULF2-specifc shRNA (Santa Cruz Biotechnology, sc-63088-V), according to the manufacturer's instructions. The infected cells were selected using 1 μg/mL of puromycin (Santa Cruz Biotechnology), and then injected into the mice to form xenograft tumors, as described above. When the tumors reached a volume of ∼500 mm, they were irradiated with 2 Gy of γ-rays. After 3 days, the mice were anesthetized, and blood was obtained through cardiac puncture and mixed with 20-fold higher volumes of RBC-lysis buffer (Intron Biotech, Seoul, Korea). Cells were collected by centrifugation (350 × *g*, 5 min), resuspended in PBS, stained with DAPI, and analyzed by confocal microscopy. A total of >3,000 stained cells were analyzed per mouse and circulating tumor cells were identified as GFP- and DAPI-positive cells [12, 38].

### Statistical analysis

All experiments were performed at least 3 times to obtain means and standard deviations. Statistical significance was defined as *P* < 0.05, which was determined using Student's-*t* tests or 1-way analysis of variance (GraphPad software, LaJolla, CA, USA).
